# A Spatio-Demographic Perspective on the Role of Social Determinants of Health and Chronic Disease in Determining a Population’s Vulnerability to COVID-19

**DOI:** 10.5888/pcd19.210414

**Published:** 2022-06-30

**Authors:** Jessica Embury, Ming-Hsiang Tsou, Atsushi Nara, Eyal Oren

**Affiliations:** 1 The Center for Human Dynamics in the Mobile Age, San Diego State University, San Diego, California; 2 Department of Geography, San Diego State University, San Diego, California; 3 School of Public Health, San Diego State University, San Diego, California

## Abstract

**Introduction:**

During the COVID-19 pandemic, health and social inequities placed racial and ethnic minority groups at increased risk of severe illness. Our objective was to investigate this health disparity by analyzing the relationship between potential social determinants of health (SDOH), COVID-19, and chronic disease in the spatial context of San Diego County, California.

**Methods:**

We identified potential SDOH from a Pearson correlation analysis between socioeconomic variables and COVID-19 case rates during 5 pandemic stages, from March 31, 2020, to April 3, 2021. We used ridge regression to model chronic disease hospitalization and death rates by using the selected socioeconomic variables. Through the lens of COVID-19 and chronic disease, we identified vulnerable communities by using spatial methods, including Global Moran *I* spatial autocorrelation, local bivariate relationship analysis, and geographically weighted regression.

**Results:**

In the Pearson correlation analysis, we identified 26 socioeconomic variables as potential SDOH because of their significance (*P* ≤ .05) in relation to COVID-19 case rates. Of the analyzed chronic disease rates, ridge regression most accurately modeled rates of diabetes age-adjusted death (*R^2^* = 0.903) and age-adjusted hospitalization for hypertensive disease (hypertension, hypertensive heart disease, hypertensive chronic kidney disease, and hypertensive encephalopathy) (*R^2^* = 0.952). COVID-19 and chronic disease rates exhibited positive spatial autocorrelation (0.304≤*I*≤0.561, 3.092≤*Z*≤6.548, 0.001≤*P*≤ .002), thereby justifying spatial models to highlight communities that are vulnerable to COVID-19.

**Conclusion:**

Novel spatial analysis methods reveal relationships between SDOH, COVID-19, and chronic disease that are intuitive and easily communicated to public health decision makers and practitioners. Observable disparity patterns between urban and rural areas and between affluent and low-income communities establish the need for spatially differentiated COVID-19 response approaches to achieve health equity.

**Summary**

**What is already known on this topic?**

Social determinants of health are positively correlated with prevalence of both COVID-19 and chronic disease. Communities characterized by low socioeconomic status and high chronic disease rates may be vulnerable to COVID-19.

**What is added by this report?**

Socioeconomic variables identified as potential social determinants of health contextualize COVID-19 health disparities by race and ethnicity. Spatial models of chronic disease and COVID-19 highlight the spatial variability of COVID-19 population vulnerability.

**What are the implications for public health practice?**

Through insight into socioeconomic conditions and chronic disease distribution, demonstrated spatial approaches support equitable COVID-19 responses at the community level.

## Introduction

As the novel coronavirus spread throughout the US in early 2020, reports of health disparity challenged claims that COVID-19 was society’s “great equalizer” (1,2). As of September 2021, non-Hispanic Black Americans, non-Hispanic American Indians, and Hispanic Americans experienced higher rates of COVID-19 infection (1.1, 1.7, 1.9 times higher, respectively), hospitalization (2.8, 3.5, 2.8 times higher, respectively), and death (2.0, 2.4, 2.3 times higher, respectively) than non-Hispanic White Americans (3). This observed health disparity stems from widespread structural discrimination and its effects on people of color.

Social determinants of health (SDOH) are socio-environmental conditions that dictate how people live and age, whereas differences in these conditions define socioeconomic status (SES) (4). Low SES is directly linked to poor health outcomes for communicable and noncommunicable diseases alike (5,6). In a study of COVID-19 outcomes in a New York City hospital, Black and Hispanic patients were more likely than White patients to present with comorbidities, such as cardiovascular disease or diabetes, that were strongly associated with mortality (7). Dr Anthony Fauci, the immunologist leading the US COVID-19 response, said that the comorbidities that negatively affect COVID-19 outcomes “relate to the social determinants of health dating back to disadvantageous conditions that some people of color find themselves in from birth” (8). Existing research confirms the associations between the disproportionate impact of COVID-19 and chronic disease in socially disadvantaged communities (6,9,10). The compounding effect of low SES, comorbidities, and COVID-19 demands immediate action to support communities vulnerable to COVID-19.

Our goal was to classify the relationships between COVID-19, chronic disease, and socioeconomic variables to promote localized public health policies. We used a spatially explicit modeling approach to meet our 2 study objectives: 1) to determine which socioeconomic variables, correlated with COVID-19 and chronic disease rates, are potential SDOH, and 2) whether spatial modeling of chronic disease rates can identify communities most vulnerable to COVID-19.

## Methods

### Study area

Our research area was San Diego County, a culturally diverse area well suited to investigation of the various effects of socioeconomic factors and chronic disease on population vulnerability to COVID-19. The county is located in southwestern California along the US–Mexico border. Its western portion is largely urban and densely populated, and its eastern portion lightly populated and rural. The county is divided into 41 subregional areas (SRAs), a geographic division frequently used to report COVID-19 and other health-related data.

### Data collection

We obtained data sets from the San Diego County Open Data Portal (11), aggregated to SRAs, containing 2017 rates for hospitalization, emergency department discharge, and death per 100,000 residents for coronary heart disease (CHD), diabetes, hypertensive diseases (hypertension, hypertensive heart disease, hypertensive chronic kidney disease, and hypertensive encephalopathy), mental illness, and pulmonary disease. We included mental illness in our study because of the toll that COVID-19 has had on mental health (12) and because of the association between mental illness, other chronic diseases, and low SES (13,14).

Socioeconomic data related to age, race and ethnicity, language, housing, income, education, and employment were retrieved from the San Diego Association of Governments (SANDAG) Data Surfer (15) and the US Census Bureau’s application programming interface (16). Data were then normalized by SRA population size or number of households. Along with socioeconomic variables, we included 4 health care access variables: health care clinics per SRA population, health care clinics per SRA square mile, hospitals per SRA population, and hospitals per SRA square mile. We calculated values for these health care access variables by using GIS analysis in ArcGIS Pro (Esri) and spatial data from SANDAG.

The County of San Diego Health and Human Services Agency provided COVID-19 rates (17) and aggregated most of the rates to SRA. However, confirmed case rates had zip code aggregations. We converted these confirmed case rates (per 100,000 residents) to the SRA extent with a 2019 population-based crosswalk from SANDAG that used dasymetric techniques to determine the proportion of residents in each zip code that live within the boundaries of an SRA. A similar crosswalk was used to aggregate the US Census Bureau socioeconomic data from census tract to SRA.

### Characterization of COVID-19 pandemic stages

**Figure 1:**
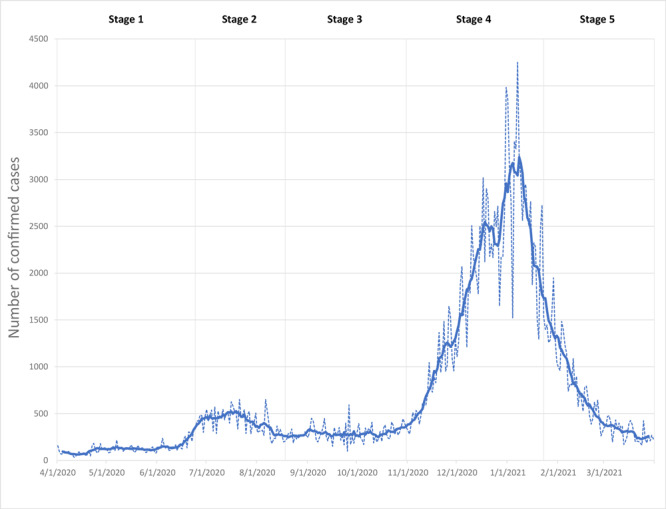
Trends in confirmed cases of COVID-19 over time, San Diego County, California, March 31, 2020, to April 3, 2021. The graph illustrates how the number of county-wide confirmed cases varied during the study period. Observed confirmed case trends were used to define 5 pandemic stages: March 31, 2020, to June 24, 2020 (Stage 1, 85 days); June 25, 2020, to August 18, 2020 (Stage 2, 54 days); August 19, 2020, to October 31, 2020 (Stage 3, 73 days); November 1, 2020, to January 23, 2021 (Stage 4, 83 days); and January 24, 2021, to April 3, 2021 (Stage 5, 69 days).

We considered 5 pandemic stages in our analysis to better understand the relationships evolving over time between COVID-19, chronic disease, and socioeconomic variables. On the basis of COVID-19 case trends in San Diego County (7-day averages), we divided the pandemic into 5 distinct stages over an approximate 12-month period, from March 31, 2020, through April 3, 2021 (18): March 31, 2020, to June 24, 2020 (Stage 1, 85 days); June 25, 2020, to August 18, 2020 (Stage 2, 54 days); August 19, 2020, to October 31, 2020 (Stage 3, 73 days); November 1, 2020, to January 23, 2021 (Stage 4, 83 days); and January 24, 2021, to April 3, 2021 (Stage 5, 69 days) (Figure 1).

**Figure 2:**
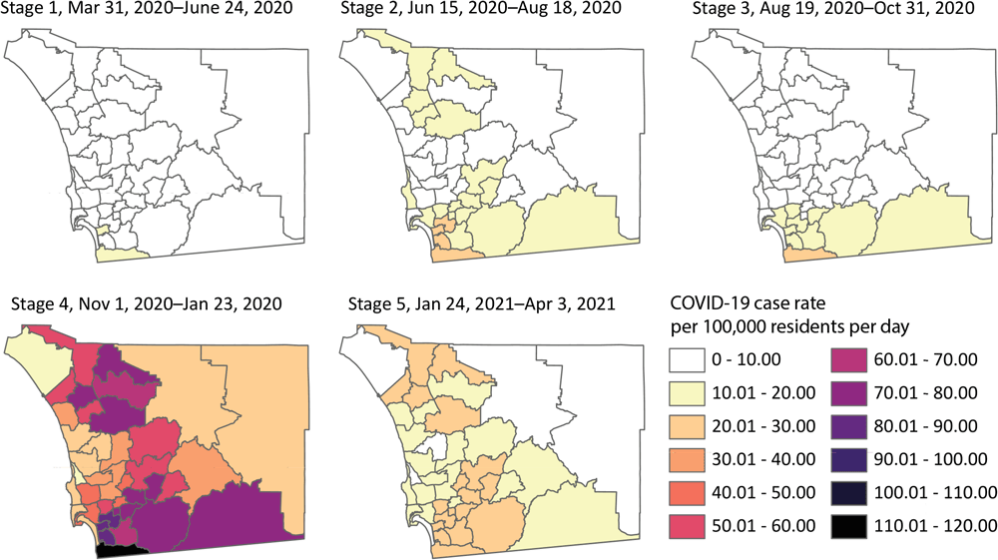
Spatial distribution of confirmed cases of COVID-19 by subregional area, San Diego County, California, March 31, 2020, to April 3, 2021. Maps show the spatial distribution of average daily COVID-19 case rates by subregional area for each of the 5 pandemic stages. Stages were determined by 7-day average case trends. All rates are per 100,000 residents.

During Stage 1, the March 19, 2020, California stay-at-home order along with local restrictions enacted from March 29 through April 4, 2020 (eg, regarding face coverings, cruise ships) kept COVID-19 rates low and stable (19). Stage 2 covered San Diego County’s first wave of increased COVID-19 rates, which followed the reopening of many of the county’s businesses, between June 13 and June 25, 2020 (the indoor operation of some business sectors reclosed on July 3, 2020) (19). Stage 3 was a period of relative stability in response to additional public health restrictions that followed the first wave. Stage 4 was characterized by a second wave of dramatic rate surges, possibly related to gatherings for the 2020 Presidential election and winter holidays. A regional stay-at-home order began on December 6, 2020, and continued through January 25, 2021 (19). Stage 5 was marked by steadily decreasing rates as the holiday season ended and county residents were vaccinated. By March 5, 2021, 1 million vaccines had been administered (19). Throughout all stages, COVID-19 confirmed case rates were highest in SRAs located in the southern portion of the county (Figure 2). Although the pandemic continues, we stopped our analysis at the end of Stage 5 to analyze and interpret existing data.

### Statistical methods

To address our first objective — to determine which socioeconomic variables, correlated with COVID-19 and chronic disease rates, were potential SDOH — we analyzed Pearson correlation coefficients, calculated with the SciPy Python package (SciPy–Python), to determine a set of potential SDOH from significant socioeconomic variables to the average confirmed daily COVID-19 case rates across the 5 pandemic stages. Socioeconomic variables were chosen for further analysis if the Pearson correlation *P* values were less than or equal to 0.05 for all stages, with 2 exceptions for variables with *P* values equal to 0.07 during 1 or 2 of the stages. The Pearson correlation coefficient is commonly used in medical research to test the strength of linear relationships between 2 variables (20). Next, we identified potentially meaningful relationships between COVID-19 and chronic disease comorbidities through a data-driven review of their Pearson correlation coefficients (18). We considered COVID-19 in the contexts of confirmed cases (total, and by race or ethnicity), total hospitalizations, and total deaths across the pandemic stages. For consistency, we selected a minimum of 1 rate, age-adjusted hospitalizations, for each of the chronic diseases.

To assess our potential SDOH, we conducted ridge regression analysis using a Python package, scikit-learn (Python), to evaluate how well the selected socioeconomic variables depicted actual distribution of COVID-19 and chronic disease. Ridge regression, a variant of linear regression, performs model regularization with a tuning parameter (α) and assigns coefficients to the explanatory variables to minimize the effects of the multicollinearity that is common among sociodemographic indicators (21). We chose the chronic disease rates with the most accurate ridge regression models for spatial analysis of COVID-19 case rates.

For our second objective, to determine whether spatial modeling of chronic disease rates can identify communities most vulnerable to COVID-19, we used 3 spatial techniques to model COVID-19 case rates and find vulnerable communities. Spatial autocorrelation (Global Moran *I*) tests of COVID-19 confirmed case rates and chronic disease rates assessed the overall appropriateness of spatial modeling. Spatial autocorrelation indicates the similarity of data values across space for a single variable, gauging whether data are clustered, dispersed, or randomly distributed (22). With local bivariate analysis and geographically weighted regression (GWR) modeling, we investigated the relationships between chronic disease rates (independent) and COVID-19 case rates (dependent). Local bivariate analysis tests for significant relationships between two variables within a spatial neighborhood (23). GWR is a regression technique that considers spatial nonstationarity and variable local relationships in the prediction model (24,25). We used Esri’s ArcGIS Pro 2.8 software (Esri) to conduct the study’s spatial analysis. Together, we synthesized the collective modeling and analysis results to propose links between COVID-19, chronic disease, and SDOH in the context of San Diego County.

## Results

### COVID-19 correlations with potential SDOH and chronic disease

From an initial data set of 79 socioeconomic variables, 26 variables were recognized as potential SDOH because of their significant linear relationships (*P* ≤ .05) to COVID-19 case rates during all 5 stages (Table 1). Two extra variables were included in the subset because at least 1 *P *value was significant (*P* ≤ .05) during 1 of 5 stages: household income of $60,000 to $75,000 during Stages 1 (*P* = .07) and 2 (*P* = .07), and household income above $200,000 for Stage 5 (*P* = .07). We discovered that some of the variables in the socioeconomic variable subset exhibited multicollinearity, such as English and Spanish as home languages, White and Hispanic race or ethnicity, and various industries of employment.

**Table 1:** Pearson Correlation Coefficients for Socioeconomic Variables^a^ and COVID-19 Daily Average Case Rates, by Stage^b^, San Diego County Subregional Areas^c^, March 31, 2020–April 3, 2021

Socioeconomic variable^d^	Stage 1	Stage 2	Stage 3	Stage 4	Stage 5
**Average number of residents per household**	0.444 (.004)	0.495 (.001)	0.412 (.007)	0.593 (<.001)	0.576 (<.001)
**Education**					
Below 9th grade	0.712 (<.001)	0.688 (<.001)	0.743 (<.001)	0.731 (<.001)	0.651 (<.001)
Bachelor’s degree or higher	−0.530 (<.001)	−0.537 (<.001)	−0.499 (<.001)	−0.680 (<.001)	−0.604 (<.001)
Master’s degree	−0.492 (.001)	−0.495 (<.001)	−0.463 (.002)	−0.625 (<.001)	−0.574 (<.001)
**Health clinics per square mile^e^**	0.614 (<.001)	0.545 (<.001)	0.553 (<.001)	0.356 (.02)	0.451 (.003)
**Language spoken at home**					
English	−0.804 (<.001)	−0.717 (<.001)	−0.734 (<.001)	−0.632 (<.001)	−0.582 (<.001)
Spanish	0.859 (<.001)	0.797 (<.001)	0.833 (<.001)	0.773 (<.001)	0.677 (<.001)
Other Indo-European language	−0.380 (.01)	−0.425 (.006)	−0.377 (.02)	−0.528 (<.001)	−0.466 (.002)
**Annual household income, $**					
Household income below the federal poverty level	0.556 (<.001)	0.434 (.005)	0.548 (<.001)	0.418 (.006)	0.305 (.05)
60,000–75,000^f^	0.285 (.07)	0.433 (.005)	0.290 (.07)	0.426 (.005)	0.587 (<.001)
>200,000^f^	−0.398 (.01)	−0.318 (.04)	−0.330 (.04)	−0.362 (.02)	−0.289 (.07)
<15,000	0.550 (<.001)	0.483 (.001)	0.543 (<.001)	0.424 (.006)	0.328 (.04)
**Households receiving cash or food assistance**	0.725 (<.001)	0.665 (<.001)	0.585 (<.001)	0.630 (<.001)	0.642 (<.001)
**Foreign-born residents**	0.584 (<.001)	0.518 (<.001)	0.527 (<.001)	0.421 (.006)	0.393 (.01)
**Households with married parents of children aged <18 years**	−0.590 (<.001)	−0.544 (<.001)	−0.550 (<.001)	−0.518 (<.001)	−0.550 (<.001)
**Residents with physical disability**	0.507 (<.001)	0.436 (.004)	0.500 (<.001)	0.586 (<.001)	0.562 (<.001)
**Residents aged 0–9 years**	0.508 (<.001)	0.563 (<.001)	0.582 (<.001)	0.636 (<.001)	0.626 (<.001)
**Race or ethnicity**					
Hispanic	0.823 (<.001)	0.808 (<.001)	0.833 (<.001)	0.806 (<.001)	0.703 (<.001)
White	−0.765 (<.001)	−0.740 (<.001)	−0.720 (<.001)	−0.648 (<.001)	−0.602 (<.001)
Other	−0.408 (.008)	−0.422 (.006)	−0.415 (.007)	−0.569 (<.001)	−0.455 (.003)
**Households with ≥1 rooms per person**	−0.710 (<.001)	−0.715 (<.001)	−0.700 (<.001)	−0.677 (<.001)	−0.640 (<.001)
**Uninsured residents**	0.511 (<.001)	0.699 (<.001)	0.638 (<.001)	0.652 (<.001)	0.686 (<.001)
**Employment, by industry**					
Management, business, science, arts	−0.564 (<.001)	−0.500 (<.001)	−0.497 (<.001)	−0.649 (<.001)	−0.564 (<.001)
Manufacturing, transportation	0.571 (<.001)	0.681 (<.001)	0.661 (<.001)	0.744 (<.001)	0.749 (<.001)
Service	0.611 (<.001)	0.506 (<.001)	0.521 (<.001)	0.553 (<.001)	0.486 (.001)
Management and administration, professional, science, waste management services	−0.449 (.003)	−0.438 (.004)	−0.392 (.011)	−0.593 (<.001)	−0.507 (<.001)

a 2019 American Community Survey 5-year estimates (16) unless otherwise noted.

b COVID-19 rates per 100,000 residents. Stages were determined by 7-day average case trends (1–5): March 31, 2020, to June 24, 2020 (Stage 1); June 25, 2020, to August 18, 2020 (Stage 2); August 19, 2020, to October 31, 2020 (Stage 3); November 1, 2020, to January 23, 2021 (Stage 4); and January 24, 2021, to April 3, 2021 (Stage 5).

c San Diego County is divided into 41 subregional areas (SRAs), a geographic division frequently used to report COVID-19 and other health-related data.

d Selected from an initial data set of 79 socioeconomic variables recognized as potential social determinants of health because of their significant linear relationships (*P* ≤ .05) to COVID-19 case rates during all 5 pandemic stages. Values are *r* (*P*) and are per 100,000.

e Values were determined by using spatial data from the San Diego Association of Governments and GIS analysis (26).

f Variable included with *P* > .05 was due to significance (*P* > .05) during other pandemic stages.

In preparation for further evaluation of the socioeconomic variable subset, we reviewed Pearson correlation coefficients for 113 chronic disease rates and 85 COVID-19–related rates and identified important relationships between COVID-19 and comorbidities. The analyzed chronic disease rates (total, age-adjusted, by sex, by race or ethnicity, by age group) included hospitalizations, emergency department discharges, and deaths related to CHD, diabetes, hypertensive disease, mental illness, and pulmonary disease with sample sizes of 30 SRAs or more. Similarly, we considered rates of COVID-19 cases, hospitalizations, and deaths (total, age-adjusted, by sex, by race or ethnicity, by age group) in sample sizes of at least 30 SRAs. Ten of the most highly correlated rates, with at least 1 for each chronic disease, were selected for regression modeling: CHD age-adjusted hospitalization, diabetes age-adjusted hospitalization, diabetes age-adjusted death, diabetes hospitalization among patients aged 65 years or older, diabetes emergency department discharge among patients aged 65 years or older, age-adjusted hospitalization for people with hypertensive disease, hospitalization of Hispanic patients with hypertensive disease, mental illness age-adjusted hospitalization, pulmonary disease age-adjusted hospitalization, and pulmonary disease hospitalization of patients aged 65 years or older (Table 2).

**Table 2:** Pearson Correlation Coefficients for 2017 Chronic Disease Rates and COVID-19 Cumulative Case Rates, by Stage^a^, San Diego County Subregional Areas^b^, March 31, 2020–April 3, 2021

Chronic disease rates^c^	Stage 1	Stage 2	Stage 3	Stage 4	Stage 5
Coronary heart disease, age-adjusted hospitalizations	0.792 (<.001)	0.746 (<.001)	0.719 (<.001)	0.745 (<.001)	0.748 (<.001)
Diabetes, age-adjusted hospitalizations	0.695 (<.001)	0.712 (<.001)	0.690 (<.001)	0.773 (<.001)	0.786 (<.001)
Diabetes, age-adjusted deaths	0.825 (<.001)	0.838 (<.001)	0.806 (<.001)	0.819 (<.001)	0.824 (<.001)
Diabetes, hospitalizations, patients aged ≥65 years	0.933 (<.001)	0.924 (<.001)	0.889 (<.001)	0.877 (<.001)	0.876 (<.001)
Diabetes, emergency department discharges, patients aged ≥65 years	0.822 (<.001)	0.798 (<.001)	0.760 (<.001)	0.749 (<.001)	0.749 (<.001)
Hypertensive diseases (hypertension, hypertensive heart disease, hypertensive chronic kidney disease, and hypertensive encephalopathy), age-adjusted hospitalizations	0.823 (<.001)	0.781 (<.001)	0.750 (<.001)	0.710 (<.001)	0.712 (<.001)
Hypertensive diseases, hospitalizations of Hispanic residents	0.887 (<.001)	0.867 (<.001)	0.833 (<.001)	0.793 (<.001)	0.790 (<.001)
Mental illness, age-adjusted hospitalizations	0.354 (.03)	0.411 (.008)	0.447 (.004)	0.571 (<.001)	0.578 (<.001)
Pulmonary disease, age-adjusted hospitalizations	0.680 (<.001)	0.657 (<.001)	0.651 (<.001)	0.704 (<.001)	0.706 (<.001)
Pulmonary disease hospitalizations, patients aged ≥65 years	0.779 (<.001)	0.771 (<.001)	0.754 (<.001)	0.810 (<.001)	0.814 (<.001)

a COVID-19 rates per 100,000 residents. Stages were determined by 7-day average case trends (1–5): March 31, 2020, to June 24, 2020 (Stage 1); June 25, 2020, to August 18, 2020 (Stage 2); August 19, 2020, to October 31, 2020 (Stage 3); November 1, 2020, to January 23, 2021 (Stage 4); and January 24, 2021, to April 3, 2021 (Stage 5).

b San Diego County is divided into 41 subregional areas (SRAs), a geographic division frequently used to report COVID-19 and other health-related data.

c All rates are per 100,000 residents. Values are *r* (*P* value).

In general, highly positive correlations were observed for chronic disease and COVID-19 rates. Key temporal patterns included:

- Decreasing correlation coefficients between COVID-19 case rates among Hispanic residents and age-adjusted hospitalizations for CHD (Stage 1: *r* = 0.80, *P* ≤ .001; Stage 5: *r* = 0.66, *P* ≤ .001), age-adjusted hospitalizations for diabetes (Stage 1: *r* = 0.79, *P* ≤ .001; Stage 5: *r* = 0.70, *P* ≤ .001), hospitalizations for diabetes among residents aged 65 years or older (Stage 1: *r* = 0.93, *P* ≤ .001; Stage 5: *r* = 0.74, *P* ≤ .001), and age-adjusted hospitalizations for hypertensive disease (Stage 1: *r* = 0.86, *P* ≤ .001; Stage 5: *r* = 0.61, *P* ≤ .001)
- High coefficients between COVID-19 death rates and diabetes death rates (eg, Stage 5, *r* = 0.86, *P* ≤ .001), emergency department discharges for patients aged 65 or older with diabetes (eg, Stage 5, *r* = 0.87, *P* ≤ .001)
- Decreasing correlation coefficients for hypertensive disease hospitalization rate and total COVID-19 case rates for Hispanic patients (Stage 1: *r* = 0.89, *P* ≤ .001; Stage 5: *r* = 0.79, *P* ≤ .001)
- Increasing correlation coefficients for age-adjusted hospitalizations for mental illness and COVID-19 case rates (Stage 1: *r* = 0.36, *P* ≤ .03; Stage 5: *r* = 0.58, *P* ≤ .001)
- High correlation coefficients between case rates among Asian residents and age-adjusted hospitalizations for pulmonary disease (eg, Stage 5: *r* = 0.89, *P* ≤ .001)
- High correlation coefficients between COVID-19 case rates among Black residents and hospitalizations for pulmonary disease among residents aged 65 years or older (eg, Stage 5: *r* = 0.71, *P* ≤ .001)

These findings suggest how the influence of medical comorbidities might have shifted as the pandemic progressed.

### Socioeconomic disease models

Ridge regression modeling showed that the potential SDOH most accurately estimated COVID-19 case rates during Stage 1 (*R^2^* = 0.893, root-mean-square deviation [RMSE] = 0.91, α = 0.01) and Stage 5 (*R^2^* = 0.875, RMSE = 2.26, α = 0.01). Elevated errors and decreased fit correspond to models of pandemic Stage 2 (*R^2^* = 0.685, RMSE = 3.43, α = 1.0) and Stage 4 (*R^2^* = 0.809, RMSE = 10.17, α = 1.0) while infection rates surged, as well as to the interim period of relative stability (Stage 3, *R^2^* = 0.789, RMSE = 1.94, α = 0.1). Ridge regression for the 10 selected chronic disease rates showed that 2 of the rates, diabetes age-adjusted death (diabetes death: *R^2^* = 0.903, RMSE = 3.15, α = 0.01) and hypertensive disease age-adjusted hospitalization (hypertensive disease hospitalization: *R^2^* = 0.952, RMSE = 21.10, α = 0.01), had *R^2^* values greater than 0.900. All other chronic disease rates had *R^2^* values below 0.810.

Although ridge regression’s regularization process limits interpretation of the effect of specific socioeconomic variables on the model, coefficients of greater magnitude (positive or negative) relative to the model run can generally be viewed as important in determining rates of COVID-19 and chronic disease. Variables corresponding to English or Spanish as home language and Hispanic ethnicity were consistently assigned coefficients of relatively high magnitude (Table 3).

**Table 3:** Ridge Regression Model Coefficients for COVID-19 Daily Average Case Rates by Stage^a^, Diabetes Age-Adjusted Death Rate^b^, and Hypertensive Disease Hospitalization Rate^b^, San Diego County Subregional Areas^c^, March 31, 2020–April 3, 2021

Socioeconomic variable^d^	Stage 1	Stage 2	Stage 3	Stage 4	Stage 5	Diabetes deaths	Hypertensive disease hospitalizations
**Average number of residents per household**	0.32	2.45	0.48	15.65	7.64	7.48	65.29
**Education**							
Below 9th grade	−11.75	−0.11	0.57	0.89	0.83	−2.02	−1,756.88
Bachelor’s degree	−6.19	−0.42	−1.36	−3.39	−19.34	−17.08	725.68
Master’s degree	−3.90	−0.02	−1.41	−0.75	−14.34	−6.19	−502.36
**Health clinics per square mile^e^**	0.26	1.13	1.00	−11.54	0.30	4.75	63.04
**Language spoken at home**							
English	−12.42	−1.81	−2.95	−3.76	−12.72	−16.87	132.43
Spanish	12.43	2.72	8.25	7.80	8.22	−9.77	803.00
Other Indo-European language	−0.03	−0.39	−0.56	−0.96	8.59	3.68	−731.42
**Annual household income, $**							
Below the federal poverty rate	2.76	−0.62	2.92	−1.34	−1.53	−12.91	−518.46
<15,000	5.61	0.11	2.09	−0.14	1.96	−3.67	561.30
60,000–75,000	3.95	0.51	0.73	1.38	36.42	21.29	−386.59
>200,000	2.97	0.99	2.56	3.24	23.98	−12.98	−428.05
**Households receiving cash or food assistance, per 100,000**	9.30	0.33	−2.63	−1.37	−1.43	28.82	248.10
**Foreign-born residents**	−7.52	0.21	−2.94	0.58	−5.96	−32.69	−210.04
**Households with married parents of children aged <18 years**	0.58	−0.18	2.15	−0.36	−26.78	13.95	275.00
**Resident with physical disability**	0.32	0.91	0.70	6.24	0.88	3.97	35.86
**Residents aged 0–9 years**	−5.01	0.14	0.18	1.31	17.13	−11.52	−179.35
**Race or ethnicity**							
Hispanic	−3.00	2.75	8.33	7.66	−1.39	−15.47	−896.04
White	2.62	−2.29	0.49	−1.24	16.73	−22.86	−522.40
Other	0.23	0.00	0.05	−0.01	1.09	0.15	−48.32
**Households with ≥1 rooms per person**	0.03	−0.53	−0.17	−2.25	−0.07	−1.11	−9.77
**Uninsured**	−7.17	0.88	1.05	1.59	20.78	−4.46	602.68
**Employment, by industry**							
Management, business, science, arts	5.88	1.71	5.95	0.91	27.65	26.45	−794.44
Manufacturing, transportation	2.40	0.61	3.77	2.01	17.01	4.43	114.61
Service	2.51	−0.61	−2.16	−1.27	11.86	−19.62	−128.65
Management and administration, professional, science, waste management services	9.67	0.46	3.27	0.46	10.39	14.19	687.10

a COVID-19 rates per 100,000 residents. Stages were determined by 7-day average case trends (1–5): March 31, 2020, to June 24, 2020 (Stage 1); June 25, 2020, to August 18, 2020 (Stage 2); August 19, 2020, to October 31, 2020 (Stage 3); November 1, 2020, to January 23, 2021 (Stage 4); and January 24, 2021, to April 3, 2021 (Stage 5).

b Chronic disease rate for 2017 per 100,000 residents. Hypertensive disease includes hypertension, hypertensive heart disease, hypertensive chronic kidney disease, and hypertensive encephalopathy.

c San Diego County is divided into 41 subregional areas (SRAs), a geographic division frequently used to report COVID-19 and other health-related data.

d Measures are per 100,000 unless otherwise indicated.

e Values were determined by using spatial data from the San Diego Association of Governments and GIS analysis (26).

### Spatial analysis of COVID-19 and chronic disease

The COVID-19 case rates in the 5 stages, diabetes deaths, and hypertensive disease hospitalizations exhibited significant positive spatial autocorrelation (Global Moran *I*) indicating that rates geographically nearby tend to be similar. Of note, the strength of spatial autocorrelation decreased for COVID-19 case rates during pandemic Stage 1 (*I* = 0.561, *z* = 6.548, *P* ≤ .001) and Stage 2 (*I* = 0.485, *z* = 5.486, *P* ≤ .001) before stabilizing during Stages 3 through 5 (0.304 ≤ *I* ≤0.347, 3.511 ≤ *z* ≤3.934, *P* ≤ .001). Spatial autocorrelation results for 2017 hypertensive disease hospitalization rates (*I* = 0.413, *z* = 4.912, *P* ≤ .001) were greater than those for the 2017 diabetes death rates (*I* = 0.345, *z* = 3.092, *P* = .002). Subsequent spatial analysis determined the accuracy with which the rate of diabetes deaths or hypertensive disease hospitalizations could be independently used to model COVID-19 case rates, thereby avoiding the multicollinearity problems inherent in the selected socioeconomic variables.

Although diabetes death rates were well estimated by ridge regression by using the potential SDOH variables, data were suppressed for most of the lightly populated (rural) SRAs. Spatial analysis with the COVID-19 case rates produced interesting results, such as a linear bivariate relationship during all stages, but the reliability of our findings is challenged by the small sample size. Visualization of diabetes deaths and COVID-19 cases with layered quantile classes separated the urban portion of the county into 3 zones: high–high positive correlations to the south, low–low positive correlations in the center, and higher than expected COVID-19 cases in the north. Also, GWR standard residuals depict the emergence of a clear spatial pattern characterized by under-predictions along major transportation corridors to the south, over-predictions in the county’s center, and under-predictions in the north.

**Figure 3:**
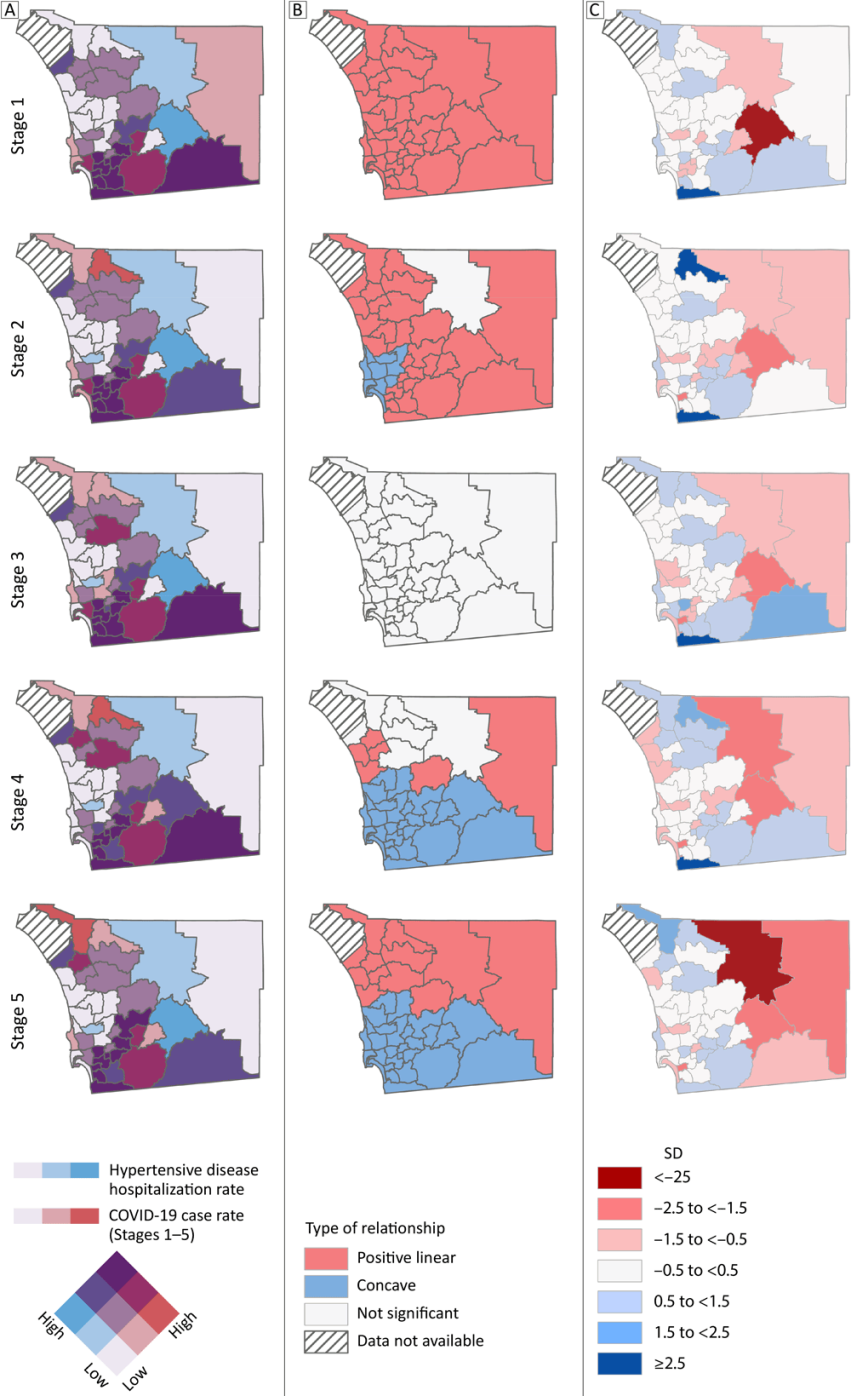
Bivariate visualizations of the age-adjusted hospitalization rate (independent) for hypertensive disease (hypertension, hypertensive heart disease, hypertensive chronic kidney disease, and hypertensive encephalopathy) and the daily average stage case rates (dependent) for COVID-19 in San Diego County subregional areas. Stages were determined by 7-day average case trends: Stage 1: March 31, 2020, to June 24, 2020; Stage 2: June 25, 2020, to August 18, 2020; Stage 3: August 19, 2020, to October 31, 2020; Stage 4: November 1, 2020, to January 23, 2021; and Stage 5: January 24, 2021, to April 3, 2021. Hospitalization rates for hypertensive disease (hypertension, hypertensive heart disease, hypertensive chronic kidney disease, and hypertensive encephalopathy) are for 2017 and consider the annual, age-adjusted rate per 100,000 residents. COVID-19 case rates consider the average daily rates per 100,000 residents for the stage. A. Layered quantile classification method for hypertensive disease hospitalization rates and the COVID-19 case rates. B. Type of local bivariate relationship for hypertensive disease hospitalization rates and COVID-19 case rates (rates not calculated for fewer than 5 events). C. Geographically weighted regression standardized residuals (prediction errors) as SDs for hypertensive disease hospitalization rates and COVID-19 case rates. Negative SD values indicate overpredicted COVID-19 case rates whereas positive SD values indicate underpredicted COVID-19 case rates.

Hypertensive disease hospitalization rates were available for all SRAs except Camp Pendleton, a military base in the northwest corner of the county. Visualization of the hypertensive disease hospitalization and COVID-19 case rates using layered quantile classification symbology showed a positive correlation, with several exceptions in northern SRAs, where northeast SRAs had higher hypertensive disease hospitalizations and northwest SRAs had higher COVID-19 cases (Figure 3A). The local bivariate analysis confirmed this observation with linear positive relationships that, in southern SRAs, shifted to concave relationships over time (Figure 3B). GWR standard residuals (prediction errors) divided the county into overpredicted SRAs to the east and underpredicted (or accurately predicted) SRAs to the west (Figure 3C). This demarcation roughly matches the county’s rural–urban divide, although rural SRAs along the US–Mexico border were also under-predicted.

## Discussion

Although the effect of socioeconomic factors on health equity is well established (5,8), spatial approaches are required to respond to known COVID-19 health disparities in regions of varied SES. We analyzed the relationships between socioeconomic variables, COVID-19, and chronic disease rates to identify a set of potential SDOH related to disproportionate disease spread. In a linear ridge regression model, variables across the categories of age, race and ethnicity, language, housing, income, education, and employment provide insight into the distribution of COVID-19. Reported health disparities related to race and ethnicity in San Diego County (27) are contextualized through the selection of related variables (eg, Hispanic ethnicity, Spanish home language) in the potential SDOH subset and their relative coefficient magnitudes during ridge regression. However, the highly related nature of the selected socioeconomic variables, such as high percentage of racial or ethnic minorities in lower-income neighborhoods (28), presents challenges to comprehensive spatial analysis.

As observed by others (7,29,30), people with preexisting chronic health conditions appear to be at increased risk of severe or fatal COVID-19 disease outcomes. As others have shown, in many cases those with an existing condition would not have died in the absence of a COVID-19 infection at the given time point (31). The strong correlations observed in our study are important in considerations related to limiting exposure for people with comorbidities, ensuring prompt vaccination to decrease biological susceptibility and providing prompt treatment if infected.

Because of the importance of comorbidities to COVID-19 outcomes and the observed correlations, we performed spatial modeling (GWR) of COVID-19 rates by using hypertensive disease hospitalization and diabetes death rates as explanatory variables. Not only can these comorbidity rates be well estimated by using the socioeconomic variables chosen to model COVID-19, but they also share similar spatial distributions to COVID-19, as determined through local bivariate analysis. Given these factors, the chronic disease rates should provide reasonable estimates of COVID-19 case rates. The GWR standard residuals indicate SRAs that have higher (underpredictions) or lower (overpredictions) COVID-19 case rates than expected by their comorbidity rates.

We propose that, in certain contexts, the GWR standard residuals highlight communities that are either notably vulnerable (underpredictions) or resilient (overpredictions) to COVID-19. When the hypertensive disease hospitalization rate is used as the explanatory variable, differences between low- and high-population SRAs become apparent, delineating the county’s rural–urban divide. When the diabetes death rate is the explanatory variable, urban subtleties reveal population vulnerabilities that can be further explained by socioeconomic variables and local area knowledge. However, because of suppressed values in the diabetes death rate data set, these findings require further investigation with additional data.

Through a spatial lens, the many interrelated factors that lead to vulnerability to COVID-19 can be better understood and clearly communicated to pandemic response decision makers and other involved planners. Spatially differentiated public health approaches are needed to overcome health disparity. The most effective policies for lightly populated communities will not work in densely populated areas. More importantly, culturally relevant and sensitive policies are needed to address COVID-19 in accordance with community demographics, preferences, and prevailing socioeconomic status. A disproportionately high number of COVID-19 cases in low-income communities might indicate low access to health care, poor communication of public health information, or unsustainable COVID-19 policies.

Our study had limitations. Data limitations posed major challenges. Health data are frequently aggregated to relatively large geographic units (ie, SRAs) and suppressed when rates are below a threshold, which ultimately resulted in a small number of large, varied areas to analyze. COVID-19 data scaled up from the zip code level are susceptible to errors related to the population-based conversion method and modifiable areal unit problem. Findings from our research are applicable only at the level of analysis and cannot be scaled down to make inferences about smaller geographic areas or individuals. Furthermore, because the temporal periods for data about the chronic disease rates (2017, annual) and COVID-19 case rates (2020–2021, 54–85 days) are not the same, uncertainties about variable correlations and temporal dependencies remain. Additional uncertainty relates to health care access in terms of who can, or will, get tested for COVID-19 or seek hospitalization and emergency services for chronic disease.

Limitations also exist in the analysis techniques used for our research. Although ridge regression regularization accommodates multicollinearity, the specific relationships between explanatory and dependent variables become obscured. In addition, our data and results suggest spatial dependency; thus, nonspatial linear models, such as ridge regression, are not reliable because they assume independence of data observations. The algorithms for neighborhood selection and prediction during the local bivariate analysis and GWR might introduce error due to varied SRA sizes. We expect that access to fine-scale data, enabling analysis with more features, would increase the accuracy of our models and enhance the overall value of the research.

Our analysis demonstrates the value of novel spatially informed approaches to COVID-19 responses and epidemiologic policy. Investigation of potential SDOH provides better understanding of the underlying reasons for COVID-19 and chronic disease distribution patterns. Socioeconomic variable analysis can help decision makers develop relevant pandemic response measures. Location unites different health and socioeconomic variables in support of clear communication about COVID-19, population vulnerability, and public health decisions. Spatial analysis is needed to develop effective policy targeted to diverse communities, such as those found in San Diego County.

Future research is needed to determine causal relationships between potential SDOH, COVID-19, and chronic disease. Access to fine-scale data and additional demographic and health care access variables, either in San Diego County or elsewhere, would permit the detailed analysis required to establish causal relationships between potential SDOH and health data. Our findings provide a basis for hypothesis formation and a framework for ongoing spatial analysis. The heterogenous nature of San Diego County is ideal for investigating how correlations differ across space and inspires ongoing research to address these differences. The promising spatial approaches discussed in this article benefit the continuing development of geographically diverse and socially equitable epidemiologic responses.
